# Secreted frizzled-related protein 2 as a context-dependent extracellular signaling hub in development, tissue remodeling, and disease

**DOI:** 10.3389/fcell.2026.1951467

**Published:** 2026-09-03

**Authors:** Xiaoping Luo, Mian Li, Yi Huang, Hong Li, Siqi You, Sichong Ren

**Affiliations:** 1 Clinical Medical College of Chengdu Medical College, Chengdu, China; 2 Department of Nephrology, School of Clinical Medicine and The First Affiliated Hospital of Chengdu Medical College, Chengdu, China

**Keywords:** extracellular signaling, fibrosis, FZD5/Ca^2+^/NFAT signaling, receptor bias, receptor switching, sFRP2, tumor microenvironment, wnt/β-catenin signaling

## Abstract

sFRP2, a member of the secreted frizzled-related protein family, is an extracellular protein characterized by a cysteine-rich domain and a netrin-like (NTR) domain. By modulating ligand availability, receptor selection, and microenvironmental signal integration, sFRP2 exerts highly context-dependent biological effects. Accumulating evidence suggests that sFRP2 should no longer be viewed merely as a WNT antagonist, but rather as an extracellular regulatory molecule that links development, tissue repair, and pathological remodeling. This review summarizes the molecular structure, expression patterns, and functional duality of sFRP2, with particular emphasis on its roles in regulating canonical and non-canonical WNT signaling, coordinating receptor bias and receptor switching, and participating in TGF-β-driven fibrosis, CXADR/NO-mediated WNT-independent growth control, endoplasmic reticulum stress-associated repair, and metabolic inflammation. We further discuss its functions within the tumor microenvironment, including the modulation of angiogenesis, cancer-associated fibroblasts, immunosuppression, and tumor cell survival. By integrating receptor bias and receptor switching with WNT-independent signaling, this review extends beyond a predominantly WNT-centered view of sFRP2 and proposes a context-stratified framework for biomarker interpretation and therapeutic targeting based on cellular origin, dominant signaling axis, disease stage, and tissue compartment.

## 1 Introduction

Secreted frizzled-related protein 2 (sFRP2) was initially characterized as a secreted Frizzled-related antagonist of WNT signaling, whereas accumulating evidence has progressively expanded this view toward a more complex role as an extracellular signaling modulator (Finch et al., 1997; Sadriev, 2025). WNT signaling is fundamental to embryonic patterning, organogenesis, and tissue homeostasis, and its dysregulation is implicated in cancer, fibrosis, and defective tissue regeneration (Liu et al., 2022; Xue et al., 2025). Importantly, the magnitude and direction of WNT signaling are determined not only by intracellular transduction machinery but also by extracellular processes governing ligand processing, spatial distribution, and receptor availability (De Almeida Magalhaes et al., 2024; Maurice and Angers, 2025). Positioned within this extracellular regulatory interface, sFRP2 can influence WNT ligand availability and receptor engagement while also participating in angiogenesis, fibrotic remodeling, tumor microenvironmental regulation, and cellular adaptation to stress (Van Loon et al., 2021; Tran et al., 2024).

Among members of the sFRP family, sFRP2 is distinguished by the breadth and apparent functional plasticity of its biological activities across developmental and reparative settings (Van Loon et al., 2021). Genetic studies in mouse embryos implicate *Sfrp2* in axial patterning and somite formation (Guan et al., 2021), whereas studies of cardiac progenitor differentiation suggest an additional role in lineage specification and maturation (Hsueh et al., 2023; Horitani and Shiojima, 2024). Following tissue injury, sFRP2 has further been associated with extracellular matrix remodeling, fibroblast activation, and scar-related responses (Cohen et al., 2024; Cheng et al., 2025). These observations collectively indicate that sFRP2 cannot be assigned a uniform biological function; rather, its effects vary according to the cellular source, tissue environment, and physiological or pathological state in which signaling occurs.

The functional diversity of sFRP2 extends to vascular and tumor-associated processes. Engagement of FZD5-associated Ca^2+^/calcineurin/NFAT signaling provides one mechanism through which sFRP2 can promote pathological angiogenesis (Wu et al., 2025), while tumor studies indicate that sFRP2-dependent vascular and immune programs may also modulate therapeutic responsiveness and provide opportunities for pharmacological intervention (Fane et al., 2020; Hsu et al., 2025). Importantly, the signaling repertoire of sFRP2 is not confined to conventional WNT-associated pathways. The identification of an sFRP2–CXADR/NO axis in retinoblastoma reveals an additional WNT-independent mode of extracellular signaling and broadens the range of membrane-associated mechanisms through which sFRP2 may influence cell behavior (Jayabal et al., 2023).

**FIGURE 1:**
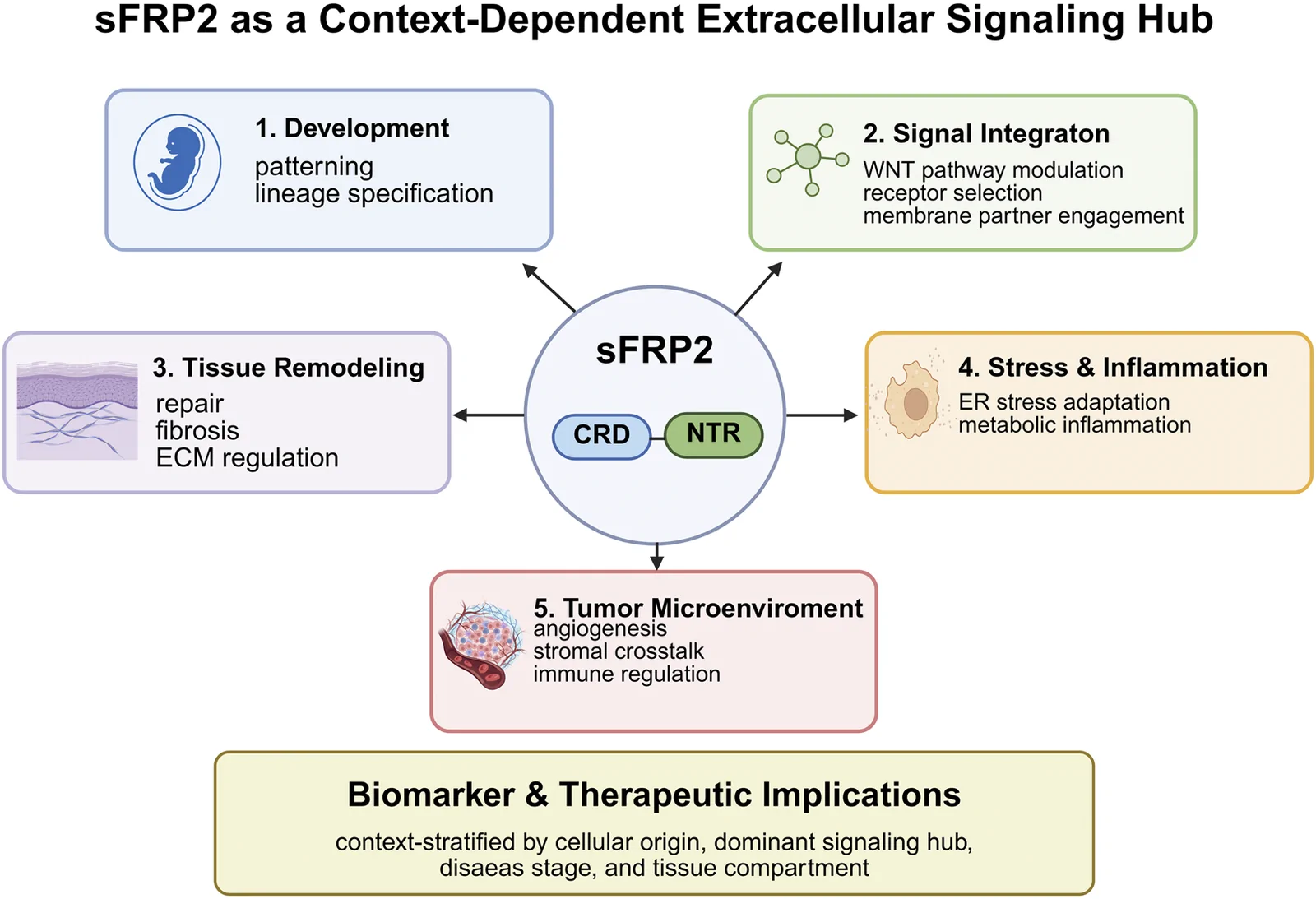
sFRP2 as a context-dependent extracellular signaling hub across development, tissue remodeling, and disease. sFRP2, characterized by cysteine-rich (CRD) and netrin-like (NTR) domains, integrates extracellular signals involved in development, WNT pathway regulation, tissue remodeling, stress and inflammatory responses, and tumor microenvironmental regulation. Its biological effects vary with ligand availability, receptor engagement, cellular origin, and microenvironmental context. This functional plasticity underlies its potential as a biomarker and therapeutic target whose interpretation requires consideration of the dominant signaling axis, disease stage, and tissue compartment. Created in BioRender. Luo, X. (2026) https://BioRender.com/iqq4xxb.

Collectively, these findings support a conceptual shift from viewing sFRP2 primarily as a soluble WNT antagonist toward considering it as a multifunctional extracellular signaling hub whose biological output is determined by signaling context. Accordingly, this review integrates current evidence regarding the roles of sFRP2 in canonical and non-canonical WNT signaling, receptor bias, receptor switching, fibrotic remodeling, ER stress-associated responses, WNT-independent CXADR/NO signaling, and tumor microenvironmental regulation. Particular emphasis is placed on reconciling the apparently divergent effects of sFRP2 across distinct cellular sources, ligand–receptor configurations, disease stages, and tissue compartments, while also identifying unresolved mechanistic questions that complicate its interpretation as a biomarker and its development as a therapeutic target. An overview of this context-dependent framework is provided in Figure 1, which positions sFRP2 as an extracellular signaling hub linking development, signal integration, tissue remodeling, stress and inflammatory responses, and tumor microenvironmental regulation.

## 2 Molecular basis and biological characteristics of sFRP2

### 2.1 Overview of the sFRP family

Secreted frizzled-related proteins (sFRPs) constitute an important class of extracellular signaling modulators that are widely involved in the molecular regulation of embryonic development, tissue homeostasis, and a broad spectrum of disease processes. As members of the Frizzled receptor-related protein family, the mammalian sFRP family primarily includes sFRP1, sFRP2, sFRP3 (FRZB), sFRP4, and sFRP5 (Table 1). A shared defining feature of these proteins is that they are secreted glycoproteins containing a highly conserved N-terminal cysteine-rich domain (CRD) and a C-terminal netrin-like (NTR) domain (Liu et al., 2022; Sadriev, 2025). The CRD is homologous to the extracellular ligand-binding region of Frizzled receptors and therefore provides the structural basis for WNT ligand recognition and receptor interaction. By contrast, the NTR domain suggests that this family is involved not only in classical ligand competition, but also in broader regulatory processes through effects on protein-protein interactions, ligand diffusion, and the extracellular matrix microenvironment (Zhang et al., 2023).

**TABLE 1:** Structural and functional characteristics of mammalian sFRP family members.

sFRP family members	Localization/secretion feature	Protein length	Main biological functions	Ref
sFRP1	Secreted extracellular glycoprotein	314	Acts as an extracellular modulator of WNT signaling and contributes to skeletal homeostasis; compared with wild-type mice, adult *Sfrp1*-deficient mice exhibit enhanced osteoblast WNT signaling and increased trabecular bone formation	(Sadriev, 2025; Bodine et al., 2004)
sFRP2	Secreted extracellular glycoprotein	295	Functions as a context-dependent extracellular signaling organizer; modulates canonical and non-canonical WNT signaling and engages distinct extracellular pathways, including BMP1-dependent collagen processing, FZD5/Ca^2+^/NFAT signaling, and CXADR/NO signaling, thereby contributing to fibrosis, angiogenesis, and tumor-associated remodeling	(Kobayashi et al., 2009; Peterson et al., 2017; Jayabal et al., 2023)
sFRP3/FRZB	Secreted extracellular glycoprotein	325	Modulates WNT signaling in skeletal development and supports articular cartilage homeostasis; compared with wild-type mice, *Frzb*-deficient mice exhibit increased WNT signaling and MMP-3 activity, accompanied by greater cartilage loss in experimental arthritis models	(Sadriev, 2025; Lories et al., 2007)
sFRP4	Secreted extracellular glycoprotein	346	Participates in WNT pathway modulation, bone morphogenesis, reproductive tissue remodeling, and vascular regulation; in endothelial models, sFRP4 induces angiostatic and apoptotic responses through suppression of NO-cGMP signaling	(Sadriev, 2025; Saran et al., 2017)
sFRP5	Secreted extracellular glycoprotein	317	Functions as a soluble WNT modulator in retinal, metabolic, inflammatory, and vascular contexts; in human endothelial cells, sFRP5 counteracts WNT5A-induced endothelial dysfunction by restoring eNOS-dependent NO production	(Sadriev, 2025; Cho et al., 2018)

**FIGURE 2:**
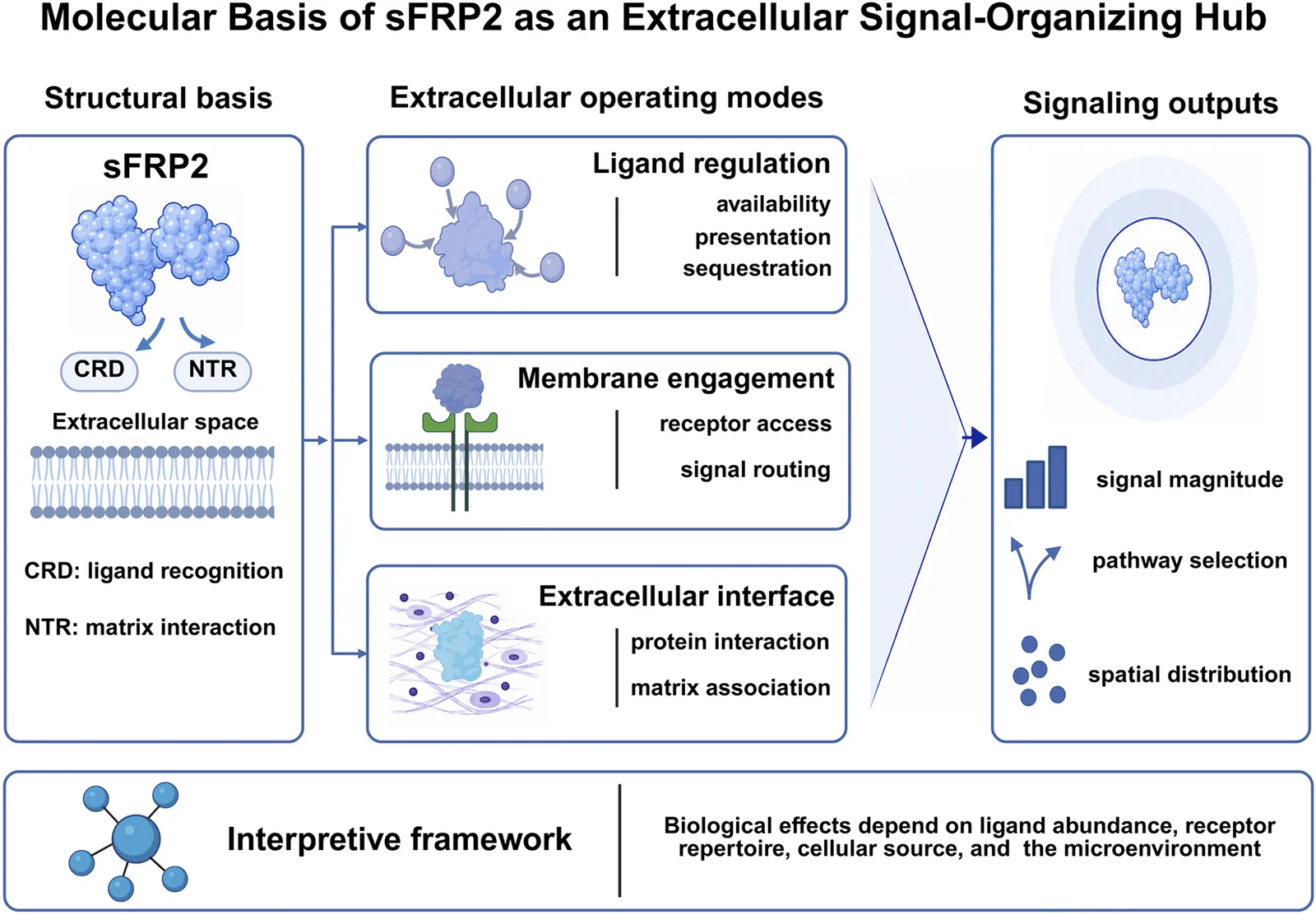
Molecular basis of sFRP2 as an extracellular signal-organizing hub. The extracellular architecture of sFRP2, comprising a CRD and a NTR domain, provides a structural basis for ligand recognition and interactions with the extracellular environment. sFRP2 can organize extracellular signaling through three interconnected operating modes: regulation of ligand availability, presentation, and sequestration; modulation of membrane engagement by influencing receptor access and signal routing; and organization of protein interactions and matrix association at the extracellular interface. Through these mechanisms, sFRP2 can modify signal magnitude, pathway selection, and the spatial distribution of signaling activity rather than exerting a uniformly inhibitory or activating effect. The resulting biological output is therefore context-dependent and is shaped by ligand abundance, receptor repertoire, cellular source, and the surrounding microenvironment. Created in BioRender. Luo, X. (2026) https://BioRender.com/licjxr9.

Early studies generally defined the sFRP family as extracellular antagonists of WNT signaling, acting through competitive binding to WNT ligands or interference with Frizzled receptor activation, thereby suppressing canonical WNT/β-catenin signal transduction. With the accumulation of subsequent evidence, however, this unidirectional inhibitory model has been substantially revised. Current data indicate that the biological effects of the sFRP family are strongly context-dependent, with their functions determined by the expression level of the individual family member, local ligand concentration, receptor repertoire composition, and the state of the tissue microenvironment. Under different conditions, sFRPs may inhibit WNT signaling, but they may also indirectly enhance specific signaling outputs by altering ligand availability, stabilizing local concentration gradients, or reshaping receptor complex formation (Xiao et al., 2015; van Loon et al., 2021). Accordingly, the sFRP family should no longer be viewed simply as a group of WNT inhibitory proteins. Aberrant expression of sFRPs has been widely observed in a variety of pathological states, including tumorigenesis and tumor progression, organ fibrosis, metabolic disorders, abnormalities in bone and cartilage development, and degenerative diseases, highlighting their role as important molecular nodes linking developmental signaling to disease-associated remodeling (Bányai and Patthy, 1999; Bodine et al., 2004; Satoh et al., 2008) (Figure 2).

### 2.2 Structure, expression, and functional duality of sFRP2

Among members of the sFRP family, sFRP2 has emerged as one of the most actively studied molecules because of its particularly complex functional spectrum and its pronounced bidirectional, context-dependent behavior. The human *SFRP2* gene is located on chromosome 4q31.3 and encodes a typical secreted sFRP family protein. Its basic structure is likewise composed of the two conserved functional domains, CRD and NTR. This molecular architecture enables sFRP2 to participate extracellularly in multiple biological processes, including ligand recognition, receptor modulation, and microenvironmental remodeling (Bovolenta et al., 2008).

The regulation of WNT signaling by sFRP2 displays marked biphasic and context-dependent properties. On the one hand, in certain tumor settings, promoter hypermethylation can silence *SFRP2* transcription, thereby relieving constraints on WNT/β-catenin signaling and supporting a tumor-suppressive role for sFRP2 (Suzuki et al., 2004; Boughanem et al., 2024). On the other hand, under specific ligand-receptor and concentration contexts, extracellular sFRP2 can potentiate canonical WNT/β-catenin signaling rather than inhibit it (von Marschall and Fisher, 2010; Liang et al., 2019; Tran et al., 2024). These findings indicate that sFRP2 is not merely antagonistic, but can shift among signal inhibition, buffering, and enhancement according to biological context.

Moreover, the functional scope of sFRP2 clearly extends beyond the canonical WNT axis itself. Previous studies have shown that sFRP2 promotes endothelial cell migration, survival, and angiogenesis through an FZD5/Ca^2+^/calcineurin/NFAT-related mechanism, suggesting that it exerts an independent pro-angiogenic effect in tumor vascular remodeling and tissue repair (Boughanem et al., 2024). In addition, sFRP2 is involved in fibrosis-associated extracellular matrix remodeling and the pathological activation of fibroblasts, and has emerged as an actionable extracellular target in multiple settings of tissue injury repair and scar formation (Cohen et al., 2024; Wang et al., 2024). Taken together, these structural and functional properties support a model in which sFRP2 acts as an extracellular signal-organizing hub, integrating ligand availability, membrane engagement, and extracellular interactions to shape signal magnitude, pathway selection, and spatial signaling distribution in a context-dependent manner (Roth et al., 2000; Suzuki et al., 2004; Zou et al., 2005). This extracellular signal-organizing model is summarized in Figure 2.

## 3 Context-dependent regulation of canonical WNT/β-catenin signaling by sFRP2

### 3.1 Core nodes of the canonical WNT/β-catenin pathway

The WNT signaling pathway is a highly conserved morphogenetic network across the animal kingdom, composed of multiple WNT ligands, receptor complexes, and intracellular transduction modules. It plays central roles in cell fate determination, polarity establishment, and migration during embryonic development, and in adult tissues it maintains stem cell and progenitor cell homeostasis. Accordingly, dysregulation of this pathway is closely associated with a broad range of diseases, including cancer and fibrosis (Maurice and Angers, 2025; Xue et al., 2025). WNT ligands must undergo Porcupine (PORCN)-mediated lipidation in the endoplasmic reticulum to acquire the hydrophobic properties required for secretion and receptor binding, after which they associate with the transport receptor Wntless/Evi (WLS) and are trafficked for intracellular transport and secretion (Nygaard et al., 2021).

The core architecture of the canonical WNT/β-catenin pathway can be summarized as follows. Upon binding of a WNT ligand to the membrane receptor Frizzled (FZD) and the co-receptor LRP5/6, Dishevelled (DVL) is recruited and activated, leading to inactivation or relocalization of the β-catenin destruction complex composed of Axin, APC, GSK3β, and CK1. This process blocks β-catenin phosphorylation and ubiquitin-mediated degradation, thereby allowing β-catenin to accumulate in the cytoplasm and translocate into the nucleus (Qi et al., 2023). Once in the nucleus, β-catenin assembles with TCF/LEF family transcription factors to form transcriptional complexes that drive the expression of a range of target genes involved in development, proliferation, and stemness. By contrast, when WNT ligand secretion is low, β-catenin is continuously phosphorylated by the destruction complex and ubiquitinated via βTrCP, thereby maintaining low basal levels and establishing threshold control over signal input (Xue et al., 2025). Canonical WNT signaling thus functions not only as a key driver of embryonic axis formation, organogenesis, and tissue regeneration, but also as an important pathogenic axis in multiple cancers and proliferative disorders, for example, when mutations in pathway components such as APC result in constitutive pathway activation (Song et al., 2024).

### 3.2 Regulation of canonical WNT/β-catenin signaling by sFRP2

The molecular basis by which sFRP2 regulates the canonical WNT pathway depends on its highly conserved structural domains and their biochemical interactions with WNT ligands and Frizzled receptors (Mafakher et al., 2025). As a member of the secreted frizzled-related protein family, mature sFRP2 consists of 295 amino acid residues and contains two core structural domains: a CRD and a NTR domain. From a structural biology perspective, the CRD shares 30%–50% sequence homology with the extracellular ligand-binding region of Frizzled receptors, whereas the NTR domain is associated with interactions with extracellular matrix and heparin-like molecules. These structural features provide the basis for its regulation of WNT ligand availability, local concentration, and signal diffusion (Bovolenta et al., 2008; Agostino and Pohl, 2020).

**FIGURE 3:**
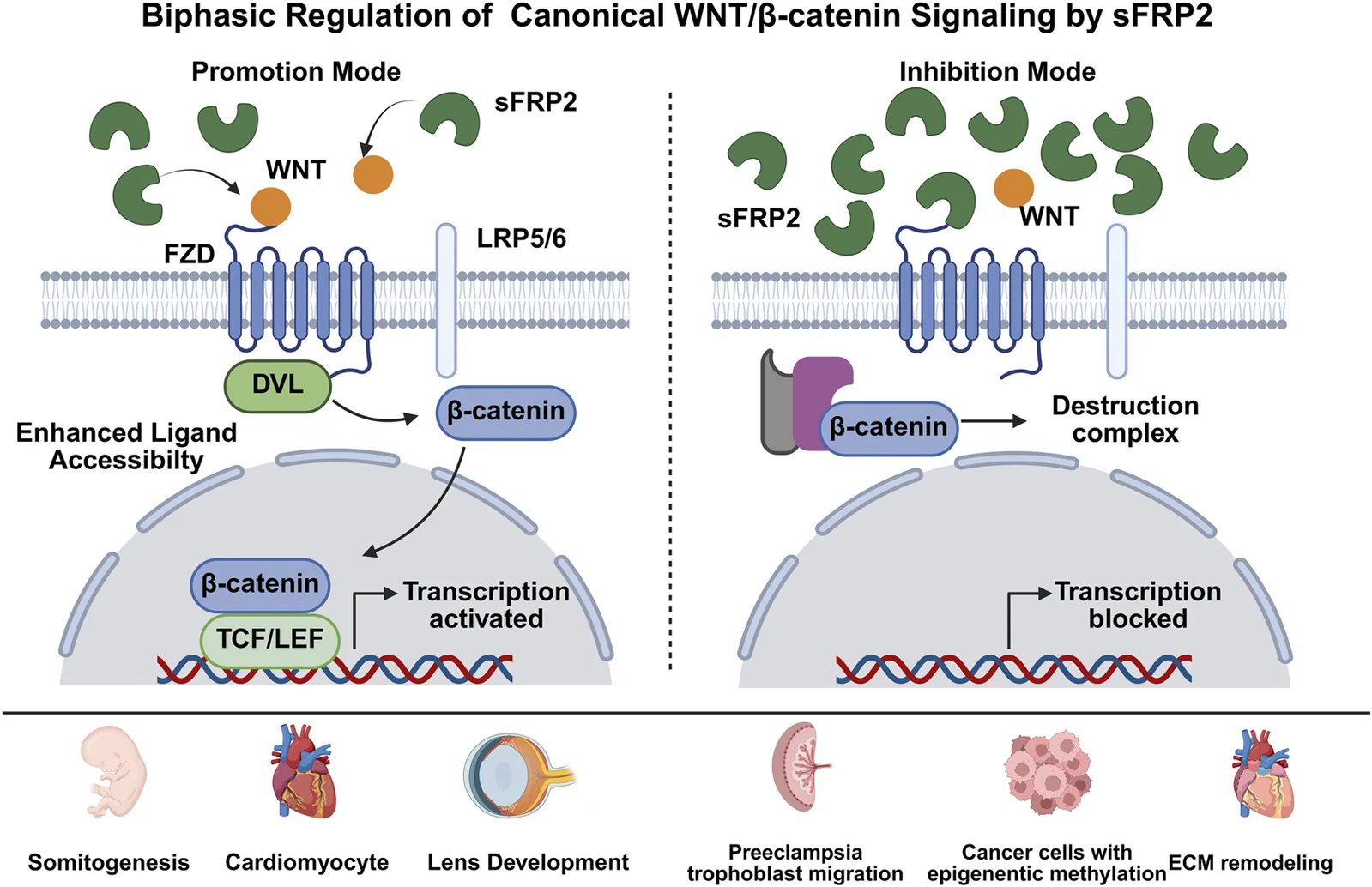
Biphasic regulation of canonical WNT/β-catenin signaling by sFRP2. sFRP2 exerts biphasic and context-dependent control over canonical WNT/β-catenin signaling. Under permissive conditions, sFRP2 can facilitate ligand presentation, stabilize extracellular WNT distribution and enhance receptor engagement, thereby promoting β-catenin accumulation and transcriptional output. In other contexts, particularly at higher local abundance or under distinct receptor compositions, sFRP2 sequesters WNT ligands or interferes with receptor complex assembly, resulting in suppression of β-catenin signaling. This bidirectional control provides a mechanistic basis for the divergent roles of sFRP2 in development, differentiation, fibrosis and tumor progression. Created in BioRender. Luo, X. (2026) https://BioRender.com/fwxztcd.

Accordingly, sFRP2 should not be simply defined as an inhibitory decoy receptor. In addition to antagonistic activity, previous studies have shown that under certain conditions it can enhance WNT3A-induced canonical β-catenin signaling and exhibits a pronounced biphasic, concentration-dependent regulatory pattern. More specifically, regulation of the canonical pathway by sFRP2 follows a finely tuned, concentration-dependent biphasic logic: at physiological or moderate concentrations, sFRP2 tends to enhance signaling strength and expand the effective range of ligand action, whereas at very high concentrations it displays the typical features of an antagonist (von Marschall and Fisher, 2010; Liang et al., 2019). Mechanistically, the agonistic activity of sFRP2 appears to be mediated, at least in part, through the regulation of WNT ligand availability and extracellular distribution rather than solely through receptor antagonism. Although earlier studies demonstrated that sFRP2 can potentiate WNT3A-induced β-catenin signaling, the underlying molecular mechanisms were not fully elucidated. More recent evidence indicates that sFRP2 can directly associate with WNT3A and promote its heparan sulfate proteoglycan-dependent retention at the cell surface, thereby facilitating ligand internalization and subsequent exosome-mediated re-secretion (Tran et al., 2024). This mechanism may enhance extracellular WNT3A availability and broaden its effective signaling range. These findings support a biphasic model in which sFRP2 can either enhance or suppress canonical WNT/β-catenin signaling depending on local sFRP2 abundance, ligand availability, and receptor context, through differential effects on ligand presentation, sequestration, and receptor engagement (Figure 3).

In developmental biology, sFRP2 contributes to morphogenesis by maintaining the directed migration of precursor cells. During mouse embryogenesis, *Sfrp1* and *Sfrp2* act synergistically, and double knockout of both genes leads to severe shortening of the thoracic region and defective somite segmentation. This phenotype is closely associated with dysregulated oscillatory expression of the Notch pathway components *Hes7* and *Lfng* (Satoh et al., 2006). In cardiac development, classic studies showed that sFRP2 regulates cardiogenesis by inhibiting WNT3A-mediated positive transcriptional feedback (Deb et al., 2008). More recent human model systems further suggest that excessive extracellular sFRP2 suppresses WNT/β-catenin output and interferes with mesodermal and cardiomyocyte differentiation, thereby supporting its role as a key extracellular regulator in myocardial development (Zhang R. et al., 2024). In addition, during visual system development, *Sfrp2* is directly regulated by the transcription factor *Pax6*, and Pax6-deficient models indicate that *Sfrp2* is one of the downstream regulators of the lens differentiation program (Shaham et al., 2009).

Once pathological remodeling is initiated, the function of sFRP2 becomes highly context-dependent and tissue-specific. In cardiovascular injury, reactivation of sFRP2 in fibroblasts within damaged regions plays a critical regulatory role. Mechanistically, this includes a WNT-independent extracellular interaction between sFRP2 and bone morphogenetic protein 1 (BMP1), through which sFRP2 enhances BMP1-dependent procollagen processing and thereby promotes collagen maturation and subsequent matrix deposition. Following myocardial infarction, sFRP2 expression is significantly positively correlated with extracellular matrix volume and the extent of cardiac fibrosis (Kobayashi et al., 2009). On the other hand, in cardiac fibroblasts, sFRP2 can partly promote cell growth, glycolysis, and lactate production through the canonical WNT/β-catenin axis, while also influencing ECM remodeling (Lin et al., 2016). Clinically, serum sFRP2 levels are positively associated with myocardial extracellular volume fraction (ECV) in patients with heart failure, whereas elevated serum sFRP2 in elderly patients with acute exacerbation of chronic heart failure is independently associated with poorer outcomes (Yang et al., 2020; Yu et al., 2025).

In a variety of epithelial malignancies, including colorectal and gastric cancers, promoter hypermethylation of *SFRP2* is relatively common. This epigenetic silencing removes restraint on β-catenin nuclear translocation and on the expression of downstream target genes such as *MYC* and *CCND1*, thereby driving cell-cycle progression from the G1 phase to the S phase (Suzuki et al., 2004; Wang et al., 2017). Consistent with its frequent epigenetic silencing in colorectal cancer (CRC), aberrant *SFRP2* methylation has also attracted considerable attention as a clinically relevant epigenetic biomarker (Boughanem et al., 2024). Stool-based detection of methylated *SFRP2* offers a non-invasive approach to CRC detection, and its diagnostic performance can be further enhanced by combination with complementary methylation markers such as *SDC2*. In the training and validation cohorts of a stool-based assay combining methylated *SFRP2* and *SDC2*, sensitivities for early-stage (stage 0–II) CRC were 89.1% and 88.5%, respectively, with specificities of 93.5% and 89.5%, respectively (Zhao et al., 2021). These findings support the potential utility of *SFRP2* methylation as a component of non-invasive early CRC screening panels. In endometriosis, aberrantly high expression of *SFRP2* resulting from promoter demethylation significantly enhances the survival and invasive capacity of ectopic lesions (Yang et al., 2023). By contrast, in placentas from patients with preeclampsia, high-level expression of sFRP2 inhibits trophoblast migration and vascular remodeling by antagonizing WNT2 signaling, thereby contributing to placental insufficiency (Lan et al., 2024). In addition, in contexts such as skin fibrosis and diabetic wound healing, dysregulated *SFRP2* expression has been linked to epidermal-dermal crosstalk, fibroblast/macrophage phenotypic transition, matrix remodeling, and healing quality (Dolivo et al., 2024; Yang et al., 2024). Overall, these studies illustrate that SFRP2 can influence disease phenotypes through context-dependent modulation of WNT signaling and related cellular programs.

## 4 Receptor-level regulation of non-canonical WNT signaling by sFRP2: Receptor bias and receptor switching

### 4.1 Receptor repertoires governing non-canonical WNT pathway selection

Non-canonical WNT signaling generally refers to branches that do not depend on β-catenin/TCF-mediated transcriptional output, primarily including the WNT/PCP (planar cell polarity) and WNT/Ca^2+^ pathways. In the WNT/PCP pathway, FZD-DVL cooperates with core PCP components such as VANGL and PRICKLE to establish intracellular asymmetry, and subsequently regulates cell polarity, collective migration, and morphogenesis through small GTPases and MAPK cascades, including RhoA, Rac, and JNK. In the WNT/Ca^2+^ pathway, specific WNT inputs induce dynamic intracellular Ca^2+^ changes and activate transcriptional programs such as NFAT, thereby influencing embryonic fate determination and tissue patterning (Yu et al., 2024; Maurice and Angers, 2025).

In addition, WNT5A is both physically and functionally coupled to the receptor tyrosine kinase-like orphan receptor ROR2, forming a prototypical non-canonical signaling axis that drives directed migration and tissue remodeling during development, such as the regulation of cell migration and proliferation during mammalian palatogenesis. In specific cellular contexts, the WNT5A-ROR2 axis can reduce β-catenin stabilization and TCF/LEF-dependent transcriptional activity, suppress transcriptional output from the canonical WNT/β-catenin pathway, and favor β-catenin-independent signaling branches by altering receptor occupancy and downstream signaling priority (Riquelme et al., 2023; Grither et al., 2024). These observations indicate that canonical and non-canonical WNT branches are not fully independent; rather, the relative allocation of signaling between them is jointly determined by ligand-receptor affinity and local effective ligand concentration, allowing the same WNT ligand in the same extracellular environment to generate signaling outputs of opposite directionality (Yu et al., 2024).

Within this receptor-dependent framework, sFRP2 appears to modulate non-canonical WNT signaling through at least two mechanistically distinct modes of receptor-level regulation. Receptor bias refers to preferential coupling of the same receptor to alternative downstream effectors, thereby altering intracellular signaling output without changing receptor identity. A representative example is the sFRP2–FZD5 axis, in which FZD5 engagement promotes Ca^2+^/calcineurin/NFATc3 signaling and favors a β-catenin-independent response (Peterson et al., 2017). Receptor switching, in contrast, involves a change in the receptor system through which ligand-dependent signaling is transmitted. During *Xenopus* gastrulation, sFRP2 redirects Wnt5a-dependent signaling from Fz7 toward Ror2, thereby enhancing Ror2-mediated non-canonical WNT activity (Brinkmann et al., 2016). These observations highlight that sFRP2 can influence non-canonical WNT signaling either by modifying downstream signaling through an engaged receptor or by altering receptor utilization itself. Notably, in several other biological settings, shifts between canonical and non-canonical WNT outputs have been documented without sufficient receptor-level evidence to distinguish between these mechanisms, indicating that the molecular basis of sFRP2-dependent pathway selection remains incompletely resolved in some contexts.

### 4.2 Crosstalk between WNT and other signaling pathways

The WNT network exhibits reproducible coupling with multiple key signaling axes. TGF-β/Smad and WNT/β-catenin pathways can cooperate at both transcriptional and protein-complex levels to promote EMT- and fibrosis-related gene programs. Studies in models such as pulmonary fibrosis suggest that TGF-β-driven phenotypic responses require participation of the β-catenin axis, while work in vascular smooth muscle cells and related systems indicates that TGF-β/Smad3 can enhance β-catenin-associated output through upstream WNT molecules (Deng et al., 2024; Trinh-Minh et al., 2024). Hippo/YAP-TAZ signaling also displays bidirectional coupling with WNT. YAP/TAZ can participate in the formation of the β-catenin destruction complex, thereby facilitating inhibitory control over β-catenin activity and reducing downstream signaling output. Conversely, WNT ligand secretion promotes the dissociation of YAP/TAZ from the destruction complex and their nuclear translocation, enhancing their own transcriptional activity and associated biological functions (Astone et al., 2024; Zhang J. et al., 2025).

Notch and WNT likewise exhibit complex synergistic and antagonistic relationships during development, cardiovascular homeostasis, and disease. In addition, signaling networks such as Hedgehog/Gli and PI3K-AKT can intersect with WNT modules in a context-dependent manner, collectively influencing cell fate, proliferation, migration, and pathological remodeling (Jing et al., 2023; Azhdari and Zur Hausen, 2025). Thus, from a pathway-level perspective, WNT signaling output is constrained by four major categories of determinants: first, ligand processing and secretion; second, the selection of cell-surface receptor and co-receptor combinations; third, the assembly and localization of intracellular hub complexes; and fourth, coupling with signaling networks such as TGF-β, Hippo/YAP, Notch, Hedgehog, and PI3K-AKT, which reprogram pathway output at both transcriptional and protein-complex levels (Wang et al., 2025). Within this framework, WNT signaling regulates cell fate, proliferation, migration, and polarity establishment during organogenesis and tissue regeneration, yet under disease conditions such as cancer and fibrosis, the same signaling modules can generate distinct pathological outputs depending on cellular and microenvironmental context.

### 4.3 sFRP2-mediated receptor bias and receptor switching

In addition to the canonical WNT/β-catenin pathway, sFRP2 also participates in the regulation of non-canonical WNT signaling. Current studies indicate that in this context, sFRP2 primarily modulates cell polarity, migration, Ca2+ homeostasis, and phenotypic transition by influencing ligand-receptor binding, receptor choice, and downstream effector coupling (Schmeckpeper et al., 2015; Brinkmann et al., 2016). In contrast to the traditional view that defines sFRP2 simply as a WNT antagonist, accumulating evidence suggests that sFRP2 can participate in the assembly and stabilization of non-canonical signaling complexes within the extracellular environment and alter the distribution of WNT signaling among distinct membrane receptors, thereby redirecting output from β-catenin-dependent branches toward PCP- or Ca^2+^-dependent pathways (Dennis et al., 1999; Grätz et al., 2023; Qian et al., 2024; Zhang Z. et al., 2024). Taken together, these observations indicate that sFRP2 can reshape non-canonical WNT signaling at both the receptor-selection and downstream-effector levels, although the receptor-level mechanism has been resolved with different degrees of certainty across experimental systems.

During *Xenopus* gastrulation, morpholino-mediated knockdown of *sfrp2* leads to defective body axis elongation accompanied by abnormal expression of the convergent extension-associated gene *papc*. Mechanistic studies have shown that sFRP2 stabilizes Wnt5a-Ror2 complexes at the cell membrane through its CRD, thereby enhancing Ror2-mediated non-canonical WNT signaling. In parallel, sFRP2 has been shown to associate with Fz7 in co-immunoprecipitation assays and to inhibit Fz7-mediated signaling, an effect that appears to involve suppression of Wnt5a-induced Fz7 receptor endocytosis. Although the precise molecular basis of this inhibitory effect remains incompletely defined, these findings support a model in which sFRP2 redirects Wnt5a-dependent signaling from Fz7 toward Ror2 (Brinkmann et al., 2016; Bous et al., 2024). These findings suggest that during development sFRP2 does not merely alter signal intensity, but rather modulates the distribution of non-canonical WNT signaling by influencing receptor switching. Studies in adult cardiac progenitor cells further show that sFRP2 promotes expression of cardiac transcription factors and differentiation toward a cardiomyocyte-like phenotype by suppressing Wnt6-associated canonical WNT transcriptional output while enhancing JNK-dependent non-canonical branch activity. Together, these results support the role of sFRP2 as an extracellular signaling organizer in developmental and regenerative contexts (Lee et al., 2006; Schmeckpeper et al., 2015). By contrast, the canonical-to-JNK shift observed in adult cardiac progenitor cells demonstrates altered pathway output but does not yet establish whether the underlying mechanism involves receptor bias or receptor switching.

**FIGURE 4:**
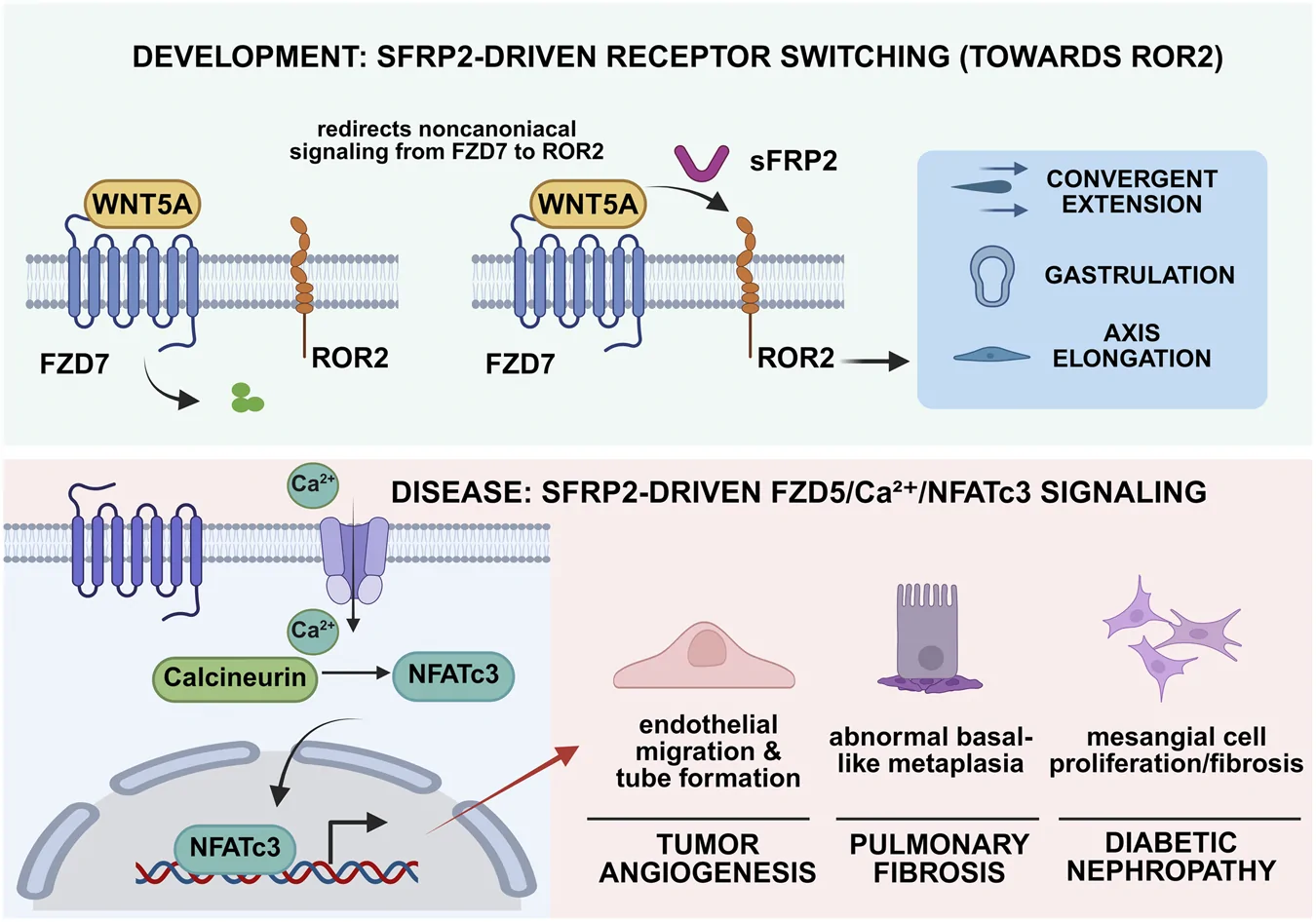
sFRP2-directed receptor switching and receptor bias in non-canonical WNT signaling. sFRP2 can reshape non-canonical WNT output through changes in receptor engagement or downstream effector coupling. During development, sFRP2 redirects Wnt5a-dependent signaling from Fz7 toward Ror2, representing receptor switching and contributing to convergent extension and axis elongation. In disease contexts, sFRP2 engages FZD5 and activates Ca^2+^-dependent calcineurin/NFAT signaling, illustrating receptor bias through preserved receptor identity with alternative downstream coupling. Other sFRP2-associated shifts in canonical versus non-canonical signaling remain mechanistically unresolved at the receptor level and are therefore presented as changes in pathway activity rather than assigned to either receptor mechanism. Together, these findings identify sFRP2 as a context-dependent determinant of receptor and pathway selection rather than a passive extracellular inhibitor. Created in BioRender. Luo, X. (2026) https://BioRender.com/bge590x.

In disease-related settings, sFRP2 can regulate disease progression through non-canonical WNT/Ca^2+^ signaling. Studies of tumor angiogenesis have shown that sFRP2 is upregulated in endothelial cells associated with multiple tumor types and promotes endothelial migration, survival, and *in vitro* tube formation (Courtwright et al., 2009; van Loon et al., 2021). Subsequent work identified FZD5 as a functional receptor for sFRP2. Upon binding to FZD5, sFRP2 induces cytosolic Ca^2+^ elevation and activates calcineurin/NFATc3 signaling; conversely, *FZD5* silencing attenuates sFRP2-induced Ca^2+^ flux, NFATc3 nuclear translocation, and tube-forming capacity. The sFRP2-FZD5-Ca^2+^-calcineurin/NFATc3 axis can therefore be regarded as a non-canonical WNT signaling pathway operative in tumor angiogenesis (Peterson et al., 2017). Through receptor bias at FZD5, sFRP2 preferentially channels downstream signaling into the Ca^2+^/calcineurin/NFATc3 cascade, thereby promoting NFATc3 nuclear translocation, endothelial activation, and tube formation and ultimately contributing to pathological angiogenesis. Together, these findings illustrate two experimentally supported modes of sFRP2-mediated receptor-level regulation: receptor switching from Fz7 toward Ror2 in developmental Wnt5a signaling and receptor bias through FZD5-dependent Ca^2+^/calcineurin/NFATc3 signaling in pathological angiogenesis (Figure 4).

This type of Ca^2+^-dependent regulation is not restricted to the tumor vascular environment. In idiopathic pulmonary fibrosis, fibroblast-derived sFRP2 acts as a downstream paracrine mediator of TGF-β1, targeting FZD5 on alveolar epithelial cells and activating NFATc3-related signaling, thereby promoting the transition of AEC2 cells from an SFTPC-positive phenotype to an aberrant KRT5/KRT17-like basalized state. Blockade of FZD5 or inhibition of the associated Ca^2+^-dependent signaling weakens this abnormal epithelial conversion (Cohen et al., 2024; Zheng et al., 2024; Zhang L. et al., 2025). These findings suggest that sFRP2 can translate fibroblast-derived signals in the fibrotic microenvironment into non-canonical WNT responses in epithelial cells, thereby contributing to pathological epithelial metaplasia.

Similar mechanisms have also been observed in metabolic and scar-forming diseases. In diabetic kidney disease, sFRP2 is upregulated in mesangial cells and promotes mesangial cell proliferation and maintenance of a fibrotic phenotype through FZD5-mediated Ca^2+^ influx and activation of the CaMKII/MEK/ERK cascade (Lv et al., 2024). Evidence from pathological scar disorders further supports a role for sFRP2 in sustaining activated fibroblast states. In hypertrophic scars, sFRP2 has been implicated in fibroblast resistance to apoptosis, whereas shRNA-mediated silencing of SFRP2 promotes apoptosis of hypertrophic scar fibroblasts (Sun et al., 2011; Chen et al., 2012). Site-dependent dysregulation of *SFRP2* expression has also been documented across distinct keloid lesional regions (Seifert et al., 2008), while transcriptomic analyses of systemic sclerosis skin have identified *SFRP2*-high fibroblast progenitor populations associated with myofibroblast differentiation (Tabib et al., 2021). In uterine scar models, locally fibroblast-derived sFRP2 enhances non-canonical WNT activity and Ca^2+^ influx, promoting fibroblast conversion toward a profibrotic phenotype. Treatment with *SFRP2*-targeting siRNA lipid nanoparticles reduces scar formation and improves pregnancy outcomes (Cheng et al., 2025). Together with evidence supporting sFRP2 as a potential antifibrotic therapeutic target (Ostrom, 2014), these observations indicate that persistent or dysregulated SFRP2 signaling can contribute to pathological fibroblast activation and maladaptive tissue remodeling across multiple organs. However, sFRP2 activity is not uniformly pathological. Under regenerative conditions, sustained local delivery of sFRP2 has been shown to enhance angiogenesis and peripheral nerve regeneration through Ca^2+^-dependent calcineurin/NFATc3 signaling (Zhang L. et al., 2025). This apparent functional divergence further underscores the context-dependent nature of sFRP2 signaling, whereby its biological consequences are shaped by tissue context, cellular state, and the spatial and temporal characteristics of sFRP2 activity. Studies in diabetic kidney disease, uterine scarring, and regenerative settings link sFRP2 to enhanced FZD5-associated or Ca^2+^-dependent non-canonical signaling, but do not fully resolve whether these effects involve altered receptor usage or downstream effector coupling. Thus, although these findings broaden the biological relevance of sFRP2-mediated non-canonical WNT signaling, its precise receptor-level mechanisms remain to be further defined.

From a translational perspective, sFRP2 is extracellular and exerts its functions through recognizable membrane receptors, making it more amenable to direct pharmacological intervention than intracellular WNT components (Larasati et al., 2022; Liu H. Y. et al., 2024). Early studies using an anti-sFRP2 monoclonal antibody showed inhibition of endothelial migration and tube formation together with reduced tumor growth in angiosarcoma and triple-negative breast cancer models relative to control-treated animals (Fontenot et al., 2013). A subsequently developed humanized anti-sFRP2 monoclonal antibody retained antitumor activity in angiosarcoma and triple-negative breast cancer models (Garcia et al., 2019), while in metastatic osteosarcoma it enhanced antitumor immune responses and improved responsiveness to PD-1 blockade (Nasarre et al., 2021). More recent work further indicates that humanized anti-sFRP2 antibodies can affect the state of tumor-associated macrophages and T cells, suggesting that blockade of sFRP2 may not only suppress angiogenesis but also reshape the tumor immune microenvironment (Hsu et al., 2025). Beyond antibody-based interventions, peptide-mediated inhibition has been investigated as an alternative approach for targeting extracellular sFRP2. Notably, an sFRP2-binding peptide (peptide II) significantly attenuated tumor growth in an orthotopic retinoblastoma model, suggesting that pharmacological interference with sFRP2-mediated signaling may provide an additional therapeutic strategy for diseases characterized by aberrant sFRP2 activity (Jayabal et al., 2023). Overall, as an extracellular regulator of the non-canonical WNT pathway, sFRP2 contributes to morphogenesis during development, lineage remodeling after injury, and pathological alterations in cell behavior in cancer and fibrosis through receptor bias and signal redistribution (Mastri et al., 2014; Kim et al., 2018).

## 5 WNT-independent signaling functions of sFRP2 in TGF-β-driven fibrotic remodeling, CXADR/NO regulation, and ER stress-associated tissue responses

### 5.1 sFRP2 as an extracellular effector of TGF-β-driven fibrotic remodeling

In fibrosis-associated diseases, sFRP2 often acts downstream of TGF-β-driven stromal activation and subsequently promotes epithelial conversion, sustained fibroblast activation, and matrix deposition. Current evidence indicates that sFRP2 is not a classical intracellular transducing component of the TGF-β/Smad pathway; rather, it is more appropriately viewed as an extracellular effector extension of this signaling axis (Henderson et al., 2020; Giarratana et al., 2024).

The relationship between sFRP2 and TGF-β/Smad-driven fibrotic remodeling is most directly illustrated in studies of pulmonary fibrosis. Investigations have shown that TGF-β1 signaling is markedly enhanced in fibroblasts from biopsies of interstitial lung disease, and *SFRP2* has been identified as a TGF-β1-inducible target gene in these fibroblasts. Inhibition of TGF-β1 signaling in fibroblasts results in a concomitant reduction in *SFRP2* expression. At the same time, fibroblast-derived sFRP2 can directly drive the transition of alveolar epithelial type II cells (AEC2s) toward a basal-like metaplastic phenotype. Thus, sFRP2 is not merely an accompanying marker of TGF-β activation, but also functions as a paracrine mediator that relays stromal TGF-β signaling into epithelial remodeling (Cohen et al., 2024). A similar regulatory pattern has been observed in uterine scar models. Studies have shown that sFRP2 remains persistently elevated in both human and murine uterine scar tissues and is predominantly enriched in uterine fibroblasts. TGF-β stimulation significantly upregulates *SFRP2* expression in human uterine fibroblasts, whereas under sustained TGF-β exposure, knockdown of *SFRP2* markedly attenuates the upregulation of fibrosis-associated molecules, including VEGFA, α-SMA, Col1A1, Col3A1, and fibrinogen, while also reducing cell migration, contractility, and myofibroblast-like transformation. These findings indicate that during uterine scar formation, sFRP2 helps maintain and reinforce the TGF-β-induced fibroblast program (Cheng et al., 2025).

In post-infarction cardiac fibrosis, the best-supported role of sFRP2 lies at the level of extracellular matrix maturation rather than as a canonical intracellular component of TGF-β/Smad signaling. Fibrogenic signaling, including TGF-β/Smad, promotes cardiac fibroblast activation and extracellular matrix gene expression, whereas extracellular sFRP2 acts at a distinct level by enhancing BMP1/Tolloid-like metalloproteinase-dependent processing of fibrillar procollagens (Massagué and Sheppard, 2023). However, bone morphogenetic protein 1 (BMP1) is not a canonical BMP signaling ligand but a secreted metalloproteinase with procollagen C-proteinase activity. Cleavage of the C-terminal propeptides from fibrillar procollagens is a key extracellular step required for collagen maturation and subsequent fibril assembly (Kessler et al., 1996). Structurally, domain-mapping experiments indicate that BMP1 binding is mediated predominantly by the CRD of sFRP2, which displays a binding affinity comparable to that of full-length sFRP2, whereas the NTR domain shows little detectable BMP1 binding. sFRP2 can also bind procollagen and enhance the association between BMP1 and procollagen, providing a mechanistic basis for its ability to increase BMP1-dependent procollagen C-proteinase activity. Consistent with this extracellular mechanism, genetic deletion of *Sfrp2* in mice impairs procollagen processing, reduces collagen deposition, attenuates post-myocardial-infarction fibrosis, and improves cardiac function relative to wild-type controls (Kobayashi et al., 2009). These findings support the sFRP2–BMP1/procollagen-processing axis as an extracellular mechanism contributing to scar maturation. However, current evidence from myocardial infarction models does not support a simple linear TGF-β–sFRP2–BMP1 signaling cascade. Instead, TGF-β/Smad-associated fibroblast activation and sFRP2-dependent extracellular matrix processing appear to represent complementary components of the fibrotic remodeling program, with the latter potentially cooperating with procollagen-processing cofactors such as PCPE1 (Zhu et al., 2019; Massagué and Sheppard, 2023).

Within the tumor microenvironment, sFRP2 is also indirectly linked to TGF-β/Smad-driven fibrotic remodeling. Single-cell studies in head and neck squamous cell carcinoma have identified a novel cancer-associated fibroblast (CAF) subset, termed *SFRP2*_CAFs, which exhibits features of both myofibroblastic CAFs (myCAFs), characterized by contractile and extracellular matrix-remodeling programs, and inflammatory CAFs (iCAFs), characterized by cytokine- and chemokine-associated inflammatory signaling. This subset is associated with poorer survival and prominent interactions with *SPP1* tumor-associated macrophages (Shi et al., 2020; Wang et al., 2024). In this context, sFRP2 more likely marks a highly activated stromal state endowed with both matrix-remodeling and inflammatory regulatory capacities, rather than defining an isolated or self-contained Smad cascade (Fang et al., 2023; Zhang Y. P. et al., 2025). In tumor tissues, sFRP2 appears to cooperate with TGF-β/Smad-driven fibrotic remodeling to promote an invasive stromal phenotype. However, whether Smad directly controls *SFRP2* transcription and how sFRP2 functions across distinct CAF subsets at different stages remain to be clarified experimentally (Chandra Jena et al., 2021; Giarratana et al., 2024; Hou, 2025).

**FIGURE 5:**
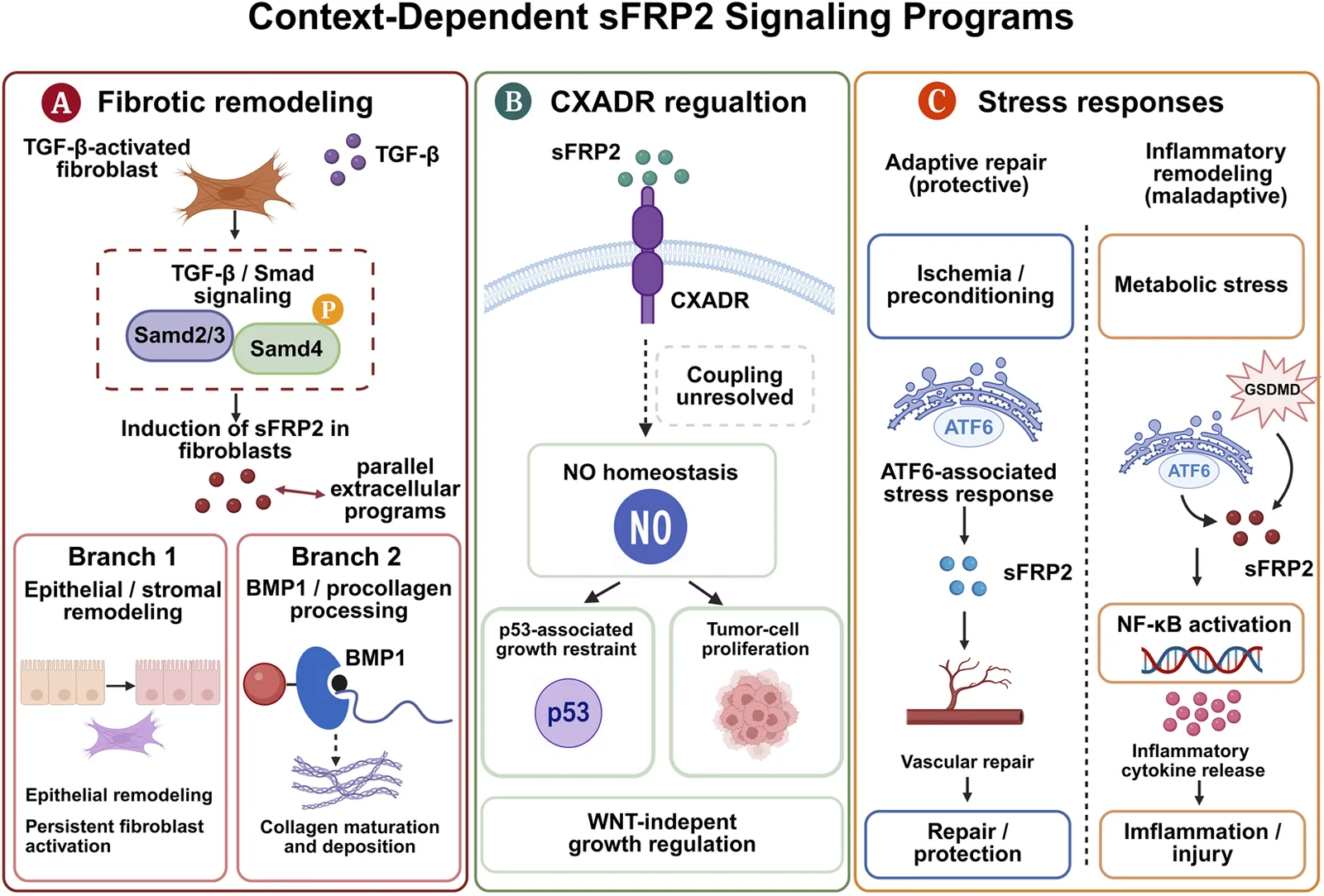
Context-dependent sFRP2 signaling in fibrotic remodeling, CXADR/NO regulation, and stress responses **(A)** TGF-β/Smad signaling induces *SFRP2* in fibroblasts, with extracellular sFRP2 contributing to epithelial/stromal remodeling and BMP1-dependent procollagen processing **(B)** In retinoblastoma, the sFRP2–CXADR axis regulates NO homeostasis and cell growth, although downstream coupling remains unresolved **(C)** In ischemic/preconditioning settings, sFRP2 is associated with ATF6-dependent adaptive repair, whereas metabolic stress engages a GSDMD–sFRP2–ATF6–NF-κB program that promotes inflammatory signaling and injury. Together, these pathways illustrate the context-dependent shift of sFRP2 between reparative and maladaptive remodeling programs. Created in BioRender. Luo, X. (2026) https://BioRender.com/v2v5xp5.

At present, sFRP2 is most appropriately defined as an extracellular extension factor of the TGF-β/Smad axis in tissue remodeling. It does not function merely as a downstream product of intracellular Smad transduction. Instead, TGF-β induces *SFRP2* expression in fibroblasts, after which sFRP2 propagates the signal into processes such as aberrant epithelial differentiation, persistent fibroblast activation, and collagen maturation and deposition (Glabman et al., 2022; Hou, 2025). This regulatory pattern has been consistently supported in pulmonary fibrosis, uterine scarring, and post-myocardial infarction remodeling. In this setting, sFRP2 functions as an extracellular mediator of TGF-β-associated fibrotic remodeling, contributing to epithelial/stromal remodeling and, through a parallel BMP1-dependent mechanism, to procollagen processing and matrix maturation. (Surana et al., 2014; Vincent and Postovit, 2017) (Figure 5A).

### 5.2 The sfrp2–cxadr/NO axis: a WNT-Independent growth-regulatory mechanism

CXADR (coxsackievirus and adenovirus receptor) is a junction-associated transmembrane protein originally characterized as a viral receptor and cell-adhesion molecule. Beyond this structural role, CXADR can organize signaling complexes at epithelial junctions. In mammary epithelial and breast cancer models, CXADR forms a MAGI-1-associated complex containing PTEN and PHLPP2, stabilizing these phosphatases and limiting AKT activation; loss of CXADR destabilizes this inhibitory signalosome and enhances TGF-β1-induced epithelial–mesenchymal plasticity (Nilchian et al., 2019; Owczarek et al., 2023). These findings establish CXADR as a membrane-associated signaling scaffold, but they do not by themselves define it as a classical autonomous signal-transducing receptor. Its receptor function therefore appears to be context-dependent and may depend on associated scaffolding or signaling proteins.

Current evidence supporting CXADR as an sFRP2-interacting membrane receptor derives primarily from retinoblastoma. Jayabal et al. combined cell-surface proteomic screening with biochemical and cell-based analyses to demonstrate a specific, CXADR-dependent interaction with sFRP2, further supported by direct high-affinity binding between the two proteins. Functional perturbation of either sFRP2 or CXADR produced reciprocal changes in cAMP/CREB/NOS2 signaling, NO production, p53-dependent growth restraint, and tumor-cell proliferation, while CXADR overexpression and sFRP2 rescue experiments further supported their functional coupling (Jayabal et al., 2023). Collectively, these findings establish CXADR as a key membrane component required for sFRP2-dependent regulation of NO homeostasis and cell growth, supporting its role as a functional receptor within a WNT-independent autocrine sFRP2–CXADR/NO axis in retinoblastoma. However, the molecular steps connecting ligand-bound CXADR to the cAMP/PKA/CREB/NOS2 pathway remain incompletely defined. Jayabal et al. proposed adenylate cyclase 9 (ADCY9) as a possible intermediary on the basis of previously reported CXADR interactome data, but this coupling was not directly tested in the sFRP2 system.

Beyond its association with NO regulation, CXADR has also been implicated in the control of PTEN/PHLPP2–AKT signaling in epithelial contexts. Previous studies in breast epithelial and breast cancer models showed that CXADR can stabilize a MAGI-1-associated inhibitory complex containing PTEN and PHLPP2, thereby restraining AKT signaling (Nilchian et al., 2019). However, these observations were obtained independently of sFRP2, and comparable evidence has not been demonstrated in retinoblastoma. In particular, it remains unknown whether sFRP2–CXADR engagement directly influences the localization, stability, or phosphatase activity of PTEN or PHLPP2 (Jayabal et al., 2023). Thus, although the PTEN/PHLPP2–AKT signalosome provides a plausible framework for CXADR-mediated signaling in other epithelial contexts, its contribution to the sFRP2–CXADR/NO axis remains to be established experimentally.

Beyond sFRP2, other members of the sFRP family have also been linked to the regulation of NO signaling in distinct biological contexts. sFRP4 has been reported to impair endothelial function and angiogenesis through suppression of the NO–cGMP pathway, whereas sFRP5 can counteract WNT5A-associated reductions in eNOS/NO signaling and improve endothelial vasodilatory responses (Saran et al., 2017; Cho et al., 2018). These observations suggest that modulation of NO signaling may represent a broader, context-dependent dimension of sFRP biology. Whether CXADR contributes to NO regulation by other sFRP family members, however, remains unknown. The identification of the sFRP2–CXADR/NO axis therefore raises the broader question of whether different sFRP proteins converge on NO-regulatory programs through distinct or shared membrane partners, an issue that remains largely unexplored.

Taken together, current evidence supports CXADR as a functional membrane partner for sFRP2 in retinoblastoma, mediating WNT-independent regulation of NO homeostasis and cell growth, although the downstream coupling mechanism remains unresolved (Figure 5B). Moreover, proposed links to ADCY9 and the PTEN/PHLPP2–AKT module have not been directly established within the sFRP2–CXADR/NO pathway (Nilchian et al., 2019; Jayabal et al., 2023). As direct validation is currently limited largely to retinoblastoma, the broader relevance of this signaling axis across other tumor types and tissue contexts remains to be determined.

### 5.3 sFRP2 and endoplasmic reticulum stress: stress adaptation, repair, and inflammation

Endoplasmic reticulum stress (ER stress) refers to a cellular stress state that arises when the protein-folding capacity of the endoplasmic reticulum becomes mismatched with secretory demand under conditions such as hypoxia, oxidative stress, disrupted calcium homeostasis, energy or glucose deprivation, lipotoxicity, or increased protein synthesis load (Acosta-Alvear et al., 2025). To restore proteostasis, cells activate the unfolded protein response (UPR), whose core consists of three canonical sensor branches: protein kinase R-like endoplasmic reticulum kinase (PERK), inositol-requiring enzyme 1α (IRE1α), and activating transcription factor 6 (ATF6). In its early phase, the UPR primarily buffers folding stress by suppressing nascent protein translation and upregulating molecular chaperones and ER-associated degradation pathways. However, when stress is prolonged or excessive, the response can shift from an adaptive program toward inflammatory amplification, cell death, and tissue injury (Matsushima et al., 2025). When misfolded proteins accumulate in the ER lumen, ATF6 dissociates from GRP78/BiP and translocates to the Golgi apparatus, where it is cleaved by the S1P and S2P proteases to release a transcriptionally active cytosolic fragment that enters the nucleus and induces the expression of molecular chaperones, folding enzymes, and other stress-responsive genes (Lei et al., 2024). In highly secretory and metabolically active tissues such as the myocardium, ATF6 not only contributes to maintenance of protein homeostasis but also translates stimuli such as ischemia and mechanical load into adaptive transcriptional programs, thereby exerting cardioprotective effects under certain conditions (Hofmann et al., 2024).

Within the myocardium, the relationship between sFRP2 and endoplasmic reticulum stress signaling is most clearly illustrated in ischemic adaptation. Acute myocardial ischemia and ischemic preconditioning (IPC) activate the unfolded protein response in cardiomyocytes, with ATF6 serving as a prominent stress-responsive branch (Doroudgar et al., 2009; An et al., 2024). This response is consistent with the broader role of ATF6 in maintaining cardiac proteostasis and supporting adaptive transcription under stress (Blackwood et al., 2020; Hofmann et al., 2024; Lei et al., 2024). In ischemic cardioprotective settings, increased sFRP2 during the third window of IPC has been associated predominantly with a CTGF-related angiogenic and arteriogenic program rather than VEGF- or FGF-dominated signaling (Doroudgar et al., 2009; Vatner et al., 2020). Moreover, 1 week after permanent coronary artery occlusion, *Sfrp2*-transgenic mice showed an approximately 49% higher left ventricular ejection fraction and a 36% smaller infarct size than wild-type littermates, and this protective phenotype was abolished by pharmacological ATF6 inhibition with AEBSF (Vatner et al., 2020). In ischemic myocardium, sFRP2 is associated with an ATF6-dependent reparative program; however, current evidence does not support a simple linear ATF6–sFRP2–CTGF cascade. ATF6 activation has been demonstrated in cardiomyocytes, whereas the principal cellular source of extracellular sFRP2 and the vascular cell populations involved in its downstream reparative effects remain unresolved (Figure 5C).

Importantly, this ischemic/pro-reparative association is mechanistically distinct from the extracellular matrix-processing activity of sFRP2 that becomes prominent during fibrotic remodeling. sFRP2 can enhance BMP1/Tolloid-like procollagen C-proteinase activity, thereby facilitating procollagen processing, collagen maturation, and matrix deposition (Kobayashi et al., 2009). The coexistence of ATF6-associated stress adaptation and BMP1-dependent matrix processing therefore illustrates the broader context dependence of sFRP2 in cardiovascular injury (Wu et al., 2020; Yin et al., 2023). This context dependence is further illustrated by findings across distinct experimental models. Genetic ablation of *Sfrp2* in mice was associated with reduced collagen deposition and attenuated post-myocardial-infarction fibrosis, whereas exogenous administration of sFRP2 in a rat myocardial infarction model reduced fibrosis and improved cardiac function (He et al., 2010). In a separate pressure-overload model, experimentally induced sFRP2 overexpression attenuated cardiac hypertrophy through modulation of WNT/β-catenin signaling (Kobayashi et al., 2009; Wei et al., 2020). These findings indicate that the biological effect of sFRP2 cannot be assigned solely on the basis of its presence or absence, but instead depends on the injury model, disease phase, dose, route and duration of exposure, and the signaling and cellular compartments engaged (Vatner et al., 2020; Yin et al., 2023).

In metabolic inflammation and diabetes-associated target-organ injury, sFRP2–ER stress coupling may intersect with inflammasome activation and pyroptotic cell death (Wang et al., 2023; Geng et al., 2024). Gasdermin D (GSDMD), a pore-forming effector of pyroptosis, provides a mechanistic link between inflammatory cell death and sFRP2-associated stress signaling. In the diabetic myocardium, Lin et al. identified a GSDMD-dependent sFRP2–ATF6–NF-κB signaling module linking inflammatory cell death to ER-stress-associated transcriptional responses. Hyperglycemia-associated oxidative stress and lipotoxicity were accompanied by GSDMD activation, enhanced sFRP2–ATF6 signaling, NF-κB activation, and increased inflammatory cytokine production. Intermittent caloric restriction suppressed this coupled pathway and attenuated myocardial inflammation and functional deterioration, whereas recombinant sFRP2 supplementation counteracted the protective effects associated with sFRP2 deficiency (Lin et al., 2025) (Figure 5C). These findings position sFRP2 at the interface between pyroptotic signaling, ER-stress regulation, and inflammatory transcription in the diabetic heart.

Beyond the diabetic myocardium, sFRP2 may intersect with GSDMD-dependent inflammatory programs in other metabolic tissues. In diabetic kidney disease, high-glucose-induced TLR4/NF-κB signaling activates the caspase-1/GSDMD pyroptotic pathway in tubular epithelial cells (Wang et al., 2019), whereas sFRP2 is independently upregulated in mesangial cells and promotes FZD5-dependent Ca^2+^/CaMKII/MEK/ERK signaling (Lv et al., 2024). Although direct coupling between sFRP2 and GSDMD has not been demonstrated in the kidney, these parallel findings suggest that sFRP2 may participate in inflammatory microenvironments in which pyroptotic signaling is active. GSDMD-mediated pyroptosis has also been implicated in sterile vascular inflammation and atherosclerotic progression (Fan et al., 2024; Huang et al., 2024), further supporting the broader relevance of this inflammatory context. Nevertheless, direct engagement of sFRP2 with GSDMD-dependent pyroptotic signaling outside the diabetic myocardium remains to be established.

### 5.4 Stage-dependent functions of sFRP2 in myocardial infarction: from ischemic repair to fibrotic remodeling

A stage-dependent interpretation is particularly useful in myocardial infarction because the dominant functional context of sFRP2 changes as ischemic injury evolves into tissue repair and subsequent scar remodeling. In ischemic and preconditioning settings, the best-supported protective evidence involves cardiomyocyte UPR/ATF6 activation together with sFRP2-associated vascular repair (Doroudgar et al., 2009; Vatner et al., 2020; An et al., 2024). Mesenchymal stem cell-derived sFRP2 has also been identified as a paracrine mediator of myocardial survival and repair (Mirotsou et al., 2007), while sFRP2 overexpression in ischemic models has been associated with reduced infarct burden and preserved ventricular function in an ATF6-dependent setting (Vatner et al., 2020). These observations support an ischemic/pro-reparative context rather than a sharply defined post-infarction time window, because the available studies differ in injury paradigm, intervention, and assessment time. At the cellular level, cardiomyocyte ATF6 activation is well supported, whereas the relative contributions of cardiomyocyte-, stromal-, and vascular-derived sFRP2 to the reparative phenotype remain incompletely defined (Wu et al., 2020).

As post-infarction remodeling progresses, the extracellular context increasingly involves fibroblast activation, matrix assembly, and scar maturation. In this setting, sFRP2 can interact with BMP1/Tolloid-like metalloproteinases and enhance procollagen C-terminal processing, thereby promoting collagen maturation and deposition; *Sfrp2* deficiency correspondingly reduces post-infarction collagen deposition and fibrosis and improves cardiac function (Kobayashi et al., 2009). This remodeling mechanism is best viewed as an extracellular matrix-processing function that is mechanistically distinct from the ATF6-associated stress-adaptation program described in ischemic cardioprotection (Blackwood et al., 2020; Vatner et al., 2020). It should likewise not be reduced to a simple linear TGF-β-sFRP2-BMP1 cascade, because current myocardial infarction studies more directly support parallel contributions of fibroblast activation and sFRP2-dependent procollagen processing within the evolving scar microenvironment (Yin et al., 2023). The transition between reparative and fibrotic states is unlikely to occur at a single discrete time point; instead, matrix stabilization and maladaptive matrix accumulation may overlap, with their relative contribution changing as healing proceeds (Yin et al., 2023).

Viewed together, the apparently discordant findings across experimental studies are more appropriately interpreted as evidence of the context-dependent actions of sFRP2 rather than as mutually exclusive results. Differences in species, injury model, disease stage, endogenous versus exogenous sFRP2 exposure, dose, spatial distribution, delivery strategy, and timing may collectively determine which downstream signaling program predominates (Wu et al., 2020; Yin et al., 2023). From a therapeutic perspective, this complexity argues against indiscriminate systemic manipulation of sFRP2. Preservation of reparative sFRP2-associated signaling may be advantageous during selected ischemic contexts, whereas selective attenuation of sustained sFRP2-BMP1-dependent matrix processing warrants evaluation once pathological fibrotic remodeling is established. These concepts remain preclinical and require direct temporal, cell-specific, and pathway-specific validation before clinical translation.

## 6 Pro-tumorigenic roles of sFRP2 in the tumor microenvironment

Within the tumor microenvironment, the functions of sFRP2 clearly extend beyond the traditional definition of a WNT antagonist. Rather, sFRP2 is more appropriately regarded as an extracellular microenvironmental effector molecule. By acting on tumor-associated endothelial cells, CAFs, myeloid immune cells, and, in some cases, tumor cells themselves, sFRP2 coordinates angiogenesis, stromal remodeling, immune exclusion, and tumor cell survival, thereby promoting tumor progression and therapeutic resistance (Wu et al., 2020; Arpinati et al., 2024; Zhang Y. P. et al., 2025).

One of the best-supported functions of sFRP2 is the promotion of pathological angiogenesis. In multiple tumor models, sFRP2 is highly expressed in tumor-associated endothelial cells and enhances endothelial migration, survival, and tube formation (Courtwright et al., 2009; Tsuruta et al., 2017; van Loon et al., 2021). FZD5 serves as its key functional receptor. Upon binding to FZD5, sFRP2 induces intracellular Ca^2+^ elevation and activates calcineurin/NFATc3 signaling, promoting NFATc3 nuclear translocation and thereby driving pro-angiogenic programs (Siamakpour-Reihani et al., 2011; Peterson et al., 2017). Silencing of FZD5 markedly attenuates these effects, indicating that the sFRP2-FZD5-Ca^2+^-calcineurin/NFATc3 axis represents the core mechanism underlying its pro-angiogenic activity (Peterson et al., 2017). This process not only enhances tumor blood supply, but may also improve tumor adaptation to hypoxic stress. In aged melanoma, sFRP2 has been identified as an age-associated angiogenic driver that can compensate for declining VEGF signaling and thereby influence responsiveness to anti-VEGF therapy (Dufourcq et al., 2002; Barandon et al., 2003; Fane et al., 2020).

Beyond its vascular effects, sFRP2 also contributes to the establishment of an immune-excluded stromal niche. Recent studies have shown that radiotherapy-induced PAI-1 can drive the conversion of perivascular cells into *SFRP2*^high CAFs through the LRP1/p65 axis (Zhang Y. P. et al., 2025). These CAFs are enriched in perivascular regions and can form a local stromal barrier that impairs recruitment and penetration of CD8^+ T cells, thereby weakening the abscopal effect and limiting responses to radioimmunotherapy (Arpinati et al., 2024; Tian and Affò, 2025; Zhang Y. P. et al., 2025). Correspondingly, blockade of sFRP2 can remodel stromal infiltration patterns and improve T-cell entry. Thus, sFRP2 is not merely an accompanying marker of CAF activation, but rather a functional driver of perivascular immunosuppressive architecture (Zhang Y. P. et al., 2025).

sFRP2 may further amplify the tumor-promoting microenvironment through interactions between CAFs and tumor-associated macrophages (TAMs). Single-cell analyses in head and neck squamous cell carcinoma have identified a distinct CAF subset, termed *SFRP2*_CAFs, which displays features of both myCAFs and iCAFs, is associated with poor prognosis, and forms prominent interaction networks with TAMs through signaling pathways such as CCL2-SPP1 (Guo and Xu, 2024; Liu C. et al., 2024; Zhang F. et al., 2024). High *SFRP2* expression therefore marks a high-risk stromal state endowed with matrix-remodeling, inflammatory-amplifying, and immunosuppressive capacities. At the same time, in triple-negative breast cancer models, humanized anti-sFRP2 monoclonal antibodies increase IFN-γ levels and alter macrophage polarization, suggesting that blockade of sFRP2 not only affects tumor vasculature but can also reshape the myeloid immune ecosystem (Vincent and Postovit, 2017; Hsu et al., 2025).

**FIGURE 6:**
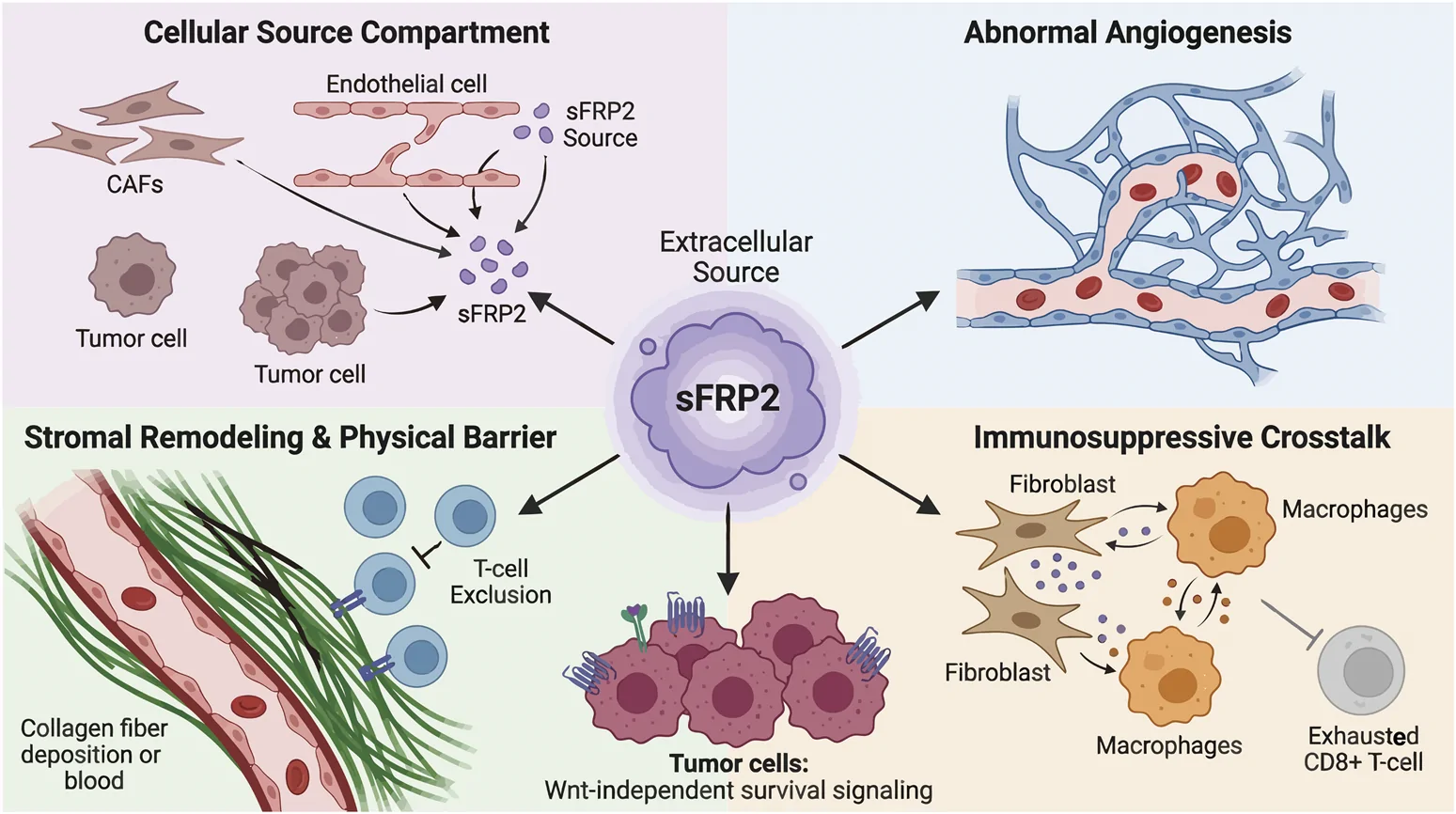
sFRP2 orchestrates multicellular pro-tumor crosstalk in the tumor microenvironment. This schematic summarizes the pro-tumor functions of sFRP2 across vascular, stromal, immune, and tumor-intrinsic compartments. Through the FZD5–Ca^2+^–calcineurin–NFAT pathway, sFRP2 enhances endothelial survival, migration, and pathological angiogenesis. In parallel, sFRP2-enriched cancer-associated fibroblasts (CAFs) promote matrix remodeling and establish immune-excluded stromal niches that restrict T cell infiltration. These stromal states further interact with tumor-associated macrophages to reinforce inflammatory and immunosuppressive signaling. In selected tumor types, sFRP2 also supports tumor cell survival through non-canonical pathways such as CXADR/NO signaling. Collectively, the figure identifies sFRP2 as an extracellular coordinator of tumor vascularization, stromal activation, immune suppression, and tumor fitness.

In certain tumors, sFRP2 also exerts tumor cell-intrinsic maintenance effects. In retinoblastoma, sFRP2 has been identified as a key autocrine factor that suppresses NO production through its cell-surface receptor CXADR, thereby relieving NO-mediated growth inhibition and sustaining tumor cell proliferation. Blockade of this axis rapidly increases NO levels and suppresses orthotopic tumor growth, revealing a potential WNT-independent sFRP2-CXADR-NO signaling axis operating within tumor cells (Jayabal et al., 2023). Taken together, the major pro-tumorigenic effects of sFRP2 in the tumor microenvironment can be summarized at four levels: promotion of tumor angiogenesis, establishment of an immune-excluded CAF barrier, enhancement of CAF-TAM tumor-promoting crosstalk, and maintenance of tumor cell-intrinsic survival in selected tumor types (Fane et al., 2020; Hsu et al., 2025; Zhang Y. P. et al., 2025). These roles collectively indicate that sFRP2 is not merely a single WNT-regulatory protein, but rather a druggable extracellular hub linking vasculature, stroma, immunity, and tumor cells themselves. Current studies have shown that monoclonal antibodies targeting sFRP2 exert antitumor, anti-angiogenic, and immune-reprogramming effects in models of angiosarcoma, triple-negative breast cancer, and metastatic osteosarcoma (Fontenot et al., 2013; Garcia et al., 2019; Nasarre et al., 2021). However, because the cellular source, dominant signaling axis, and functional intensity of sFRP2 remain strongly tumor type-dependent, future studies will need to further define the most appropriate interventional settings on the basis of cellular lineage, spatial distribution, and treatment context stratification (Liu C. et al., 2024; Zhang Y. P. et al., 2025) (Figure 6).

## 7 Discussion and future perspectives

Because sFRP2 is localized extracellularly, it can be detected in body fluids and tissue compartments. At the same time, it lies at the intersection of WNT ligand delivery, FZD5/NFAT signaling, ECM processing, and fibroblast-epithelial/immune cell interactions. It therefore holds promise both as an indicator of disease activity and as a potential therapeutic target (Yang et al., 2016; Sun et al., 2019; Zhao et al., 2020). In CRC, systematic evaluations of fecal *SFRP2* methylation have demonstrated relatively high specificity and favorable overall diagnostic performance for CRC detection (Yang et al., 2016; Raut et al., 2020). When *SFRP2* and *SDC2* methylation are combined, sensitivity for advanced adenoma and CRC detection is higher than that achieved by either methylation marker alone in plasma, and similarly high sensitivity for early-stage CRC has been reproduced in stool-based testing (Zhao et al., 2020; Zhao et al., 2021). More recently, a multicenter case-control study showed that a stool-based *SDC2/SFRP2/TFPI2* methylation panel achieved an AUC of 0.9399 for CRC detection, compared with 0.7532 for FOBT and 0.6732 for serum CEA (Li et al., 2026), further supporting the inclusion of *SFRP2* in multi-marker screening panels rather than its use as an isolated biomarker. Whole-blood analyses have further shown that *SFRP2* promoter methylation can predict CRC stage, lymph-node invasion, and recurrence (Boughanem et al., 2024), while earlier work also documented stage-related variation in *SFRP2* methylation across tissue, fecal, and blood samples (Sui et al., 2016).

However, the biomarker significance of sFRP2 is clearly context-dependent. In CRC epithelial cells, *SFRP2* is commonly silenced by methylation, whereas in solid tumors such as head and neck squamous cell carcinoma, CAF subsets with high *SFRP2* expression are associated with poorer survival and enhanced interaction with *SPP1* macrophages (Liu C. et al., 2024; Wang et al., 2024). In the setting of heart failure, circulating sFRP2 levels also show inconsistent directional changes across studies: some have reported reduced serum sFRP2 in heart failure (HF) accompanied by type 2 diabetes mellitus (T2DM), whereas others have found that elevated serum sFRP2 predicts worse mortality or rehospitalization outcomes in elderly patients with acute exacerbation of chronic heart failure (Cao et al., 2021; Yu et al., 2025). Therefore, if sFRP2 is to be advanced as a clinical biomarker, the type of specimen measured must be clearly defined, and its direction of change and expression threshold must be interpreted within specific disease contexts (Wang et al., 2017).

As an extracellular therapeutic target, sFRP2 offers the advantage that intervention does not require transmembrane delivery into cells. Moreover, binding of sFRP2 to FZD5 can activate the Ca^2+^/calcineurin/NFATc3 axis, directly participating in tumor angiogenesis and pathological stromal remodeling, thereby providing a clear pharmacological target for antibodies, nucleic acid-based therapies, and local delivery strategies (Tsuruta et al., 2014; Peterson et al., 2017). FZD5 is likely the key receptor mediating sFRP2-induced endothelial tube formation, Ca^2+^ flux, and NFATc3 activation (Peterson et al., 2017). On this basis, an early anti-sFRP2 monoclonal antibody suppressed tumor growth in angiosarcoma and triple-negative breast cancer models (Fontenot et al., 2013), whereas a subsequently developed humanized anti-sFRP2 antibody reduced tumor burden relative to IgG1-treated controls and increased tumor-cell apoptosis *in vivo* without evident immunogenicity (Garcia et al., 2019). In metastatic osteosarcoma models, humanized anti-sFRP2 treatment enhances T-cell proliferation, reduces CD38/PD-1 expression, and improves responsiveness to anti-PD-1 therapy (Nasarre et al., 2021). Separately, in TNBC models, sFRP2 blockade increases IFN-γ and the M1/M2 macrophage ratio, indicating an additional immune-reprogramming effect (Hsu et al., 2025). In addition, studies on uterine scarring have shown that lipid nanoparticle-mediated delivery of siRNA targeting *SFRP2* reduces scar formation and improves pregnancy outcomes, supporting the applicability of extracellular sFRP2-targeted interventions to fibrosis and aberrant repair disorders (Wei et al., 2020; Cheng et al., 2025).

The greatest limitation of sFRP2, however, stems from this same biological pleiotropy. On the one hand, sFRP2 functions as an enhancer of procollagen C-proteinase activity in BMP1/Tolloid-like metalloproteinase systems, and genetic deletion of *Sfrp2* in mice attenuates post-myocardial-infarction fibrosis and improves cardiac function relative to wild-type controls (Kobayashi et al., 2009). On the other hand, during specific windows of ischemic stress or repair, upregulation of sFRP2 can promote angiogenesis, reduce infarct size, and improve ejection performance through ATF6-CTGF-related programs, indicating that blockade of sFRP2 may not be beneficial in all cardiac disease settings (Mirotsou et al., 2007; Vatner et al., 2020). At the same time, sFRP2 can both enhance WNT3A signaling and promote the formation of WNT-sFRP2 heteromeric complexes that increase exosome-mediated re-secretion through HSPG-related mechanisms. Sustained systemic inhibition of sFRP2 may therefore interfere with normal ligand transport processes involved in tissue repair, regeneration, and morphogenesis (Mii and Takada, 2020; Gross, 2021; Tran et al., 2024).

Accordingly, future studies should evaluate the therapeutic potential of sFRP2 within a stratified framework that incorporates cellular origin, disease stage, tissue compartment, and pathway bias. Only through such context-specific stratification will it be possible to define the most appropriate interventional settings for targeting sFRP2 in cancer and fibrosis. The principal translational applications and their corresponding limitations and challenges are summarized in Table 2.

**TABLE 2:** Translational applications of SFRP2 as a biomarker and therapeutic target: Current evidence, limitations, and challenges.

Strategy/agent	Modality/approach	Main functionality	Models/contexts	Limitations/challenges	Ref
Fecal *SFRP2* methylation	DNA methylation marker	Supports early CRC and advanced adenoma screening; best used in multi-marker panels	Stool DNA testing	Single-marker performance is context-dependent; validated multi-marker panels improve interpretability	(Yang et al., 2016; Raut et al., 2020; Zhao et al., 2021)
*SDC2/SFRP2/TFPI2* panel	Multi-gene methylation panel	Improves detection of CRC and advanced adenomas compared with single-marker testing	CRC screening cohorts	Evidence is concentrated in CRC cohorts; generalizability across populations and settings remains to be established	(Li et al., 2026)
Blood *SFRP2* methylation	Circulating epigenetic marker	May reflect CRC stage, lymph node involvement, recurrence risk, and follow-up value	Blood samples from CRC patients	Stage- and specimen-dependent variation complicates cross-study interpretation and threshold definition	(Sui et al., 2016; Boughanem et al., 2024)
Anti-sFRP2 monoclonal antibody	Antibody blockade	Inhibits endothelial migration, tube formation, and pathological angiogenesis via the FZD5/Ca^2+^/NFAT axis	Angiosarcoma; TNBC	Evidence remains preclinical; systemic blockade may affect reparative sFRP2 signaling, while target selectivity and unintended effects require further characterization	(Fontenot et al., 2013; Peterson et al., 2017; Garcia et al., 2019)
Humanized anti-sFRP2 antibody	Humanized antibody	Reduces tumor burden and angiogenesis; may also remodel the immune microenvironment	Angiosarcoma; TNBC; osteosarcoma	Preclinical efficacy is tumor-type dependent; clinical safety and effects of sustained systemic blockade remain unresolved	(Garcia et al., 2019; Nasarre et al., 2021; Hsu et al., 2025)
Anti-sFRP2 + anti-PD-1	Combination therapy	Enhances antitumor T-cell responses and may improve sensitivity to immune checkpoint blockade	Metastatic osteosarcoma	Evidence is limited to metastatic osteosarcoma models; applicability to other tumors remains uncertain	(Nasarre et al., 2021)
sFRP2-CXADR/NO blockade	Peptide or pathway blockade	Increases NO production and suppresses WNT-independent tumor growth	Retinoblastoma xenografts	Validation is largely confined to retinoblastoma; downstream CXADR coupling and broader relevance remain unresolved	(Jayabal et al., 2023)
*SFRP2* siRNA-LNP	Local nucleic-acid delivery	Reduces fibroblast activation and scar formation by locally silencing *SFRP2*	Uterine scar models	Evidence is limited to local uterine scar models; durability and broader antifibrotic applicability require evaluation	(Cheng et al., 2025)
FZD5-axis inhibition	Receptor-axis intervention	Attenuates sFRP2-induced Ca^2+^ signaling, NFAT activation, angiogenesis, and fibrotic conversion	Tumor angiogenesis; IPF; DKD	Receptor-level evidence varies across models; pathway selectivity and receptor mechanisms remain incompletely defined	(Peterson et al., 2017; Cohen et al., 2024; Lv et al., 2024)

## 8 Conclusion

sFRP2 regulates extracellular signaling in a context-dependent manner during development, tissue repair, fibrotic remodeling, metabolic inflammation, and tumor progression. This review supports a revised conceptual framework in which sFRP2 functions not solely as an extracellular antagonist of WNT signaling, but as a multifunctional regulator that modulates canonical and non-canonical WNT pathways, influences receptor selection, regulates extracellular matrix maturation, and participates in WNT-independent signaling mechanisms, including the CXADR/NO axis and ER stress-associated inflammatory responses. Through these mechanisms, sFRP2 contributes to the integration of ligand availability, receptor engagement, stromal activation, vascular remodeling, and immune regulation within distinct tissue microenvironments.

The functional consequences of sFRP2 activity are determined by cellular origin, receptor composition, disease stage, tissue compartment, and local microenvironmental conditions. These variables may account for the divergent roles of sFRP2 in adaptive repair, pathological fibrosis, metabolic injury, and tumor progression. Future studies should define the dominant receptor axis, temporal expression pattern, cellular source, and downstream signaling output of sFRP2 in specific disease contexts. Such mechanistic stratification will be required to clarify the clinical value of sFRP2 as a biomarker and to identify pathological settings in which therapeutic targeting of extracellular sFRP2 may provide translational benefit.
